# Data-Driven Synthetic Cell Factories Development for Industrial Biomanufacturing

**DOI:** 10.34133/2022/9898461

**Published:** 2022-06-15

**Authors:** Zhenkun Shi, Pi Liu, Xiaoping Liao, Zhitao Mao, Jianqi Zhang, Qinhong Wang, Jibin Sun, Hongwu Ma, Yanhe Ma

**Affiliations:** ^1^Key Laboratory of Systems Microbial Technology, Tianjin Institute of Industrial Biotechnology, Chinese Academy of Sciences, Tianjin 300308, China; ^2^National Technology Innovation Center of Synthetic Biology, Tianjin 300308China

## Abstract

Revolutionary breakthroughs in artificial intelligence (AI) and machine learning (ML) have had a profound impact on a wide range of scientific disciplines, including the development of artificial cell factories for biomanufacturing. In this paper, we review the latest studies on the application of data-driven methods for the design of new proteins, pathways, and strains. We first briefly introduce the various types of data and databases relevant to industrial biomanufacturing, which are the basis for data-driven research. Different types of algorithms, including traditional ML and more recent deep learning methods, are also presented. We then demonstrate how these data-based approaches can be applied to address various issues in cell factory development using examples from recent studies, including the prediction of protein function, improvement of metabolic models, and estimation of missing kinetic parameters, design of non-natural biosynthesis pathways, and pathway optimization. In the last section, we discuss the current limitations of these data-driven approaches and propose that data-driven methods should be integrated with mechanistic models to complement each other and facilitate the development of synthetic strains for industrial biomanufacturing.

## 1. Introduction

In the last two decades, high-throughput omics technologies enabled by sequencing have revolutionized the way we study biological systems [1]. These technologies enable the systematic experimental measurement at various molecule levels, at different time scales, and from single cells to a community of organisms. The vast amounts of data obtained from these measurements are turning biosciences into a data-centric science subject [2, 3]. A big challenge is how to effectively convert the big data into useful knowledge to help us better understand the genotype-phenotype relationship, the organizing principles of complex biosystems, and subsequently design/engineer new biosystems for medical, agricultural, and industrial applications [3].

Design and engineering of biosystems with new abilities is the key objective of synthetic biology, an emerging multidisciplinary research area that integrates a broad range of methodologies from various disciplines such as mathematics, chemistry, and computer science. Synthetic biology makes use of advanced new technologies to modify or create new biological parts, multienzyme bioconversion pathways, and artificial cell factories [4–7]. One important application of synthetic biology is to produce various biochemicals, biofuels, and biomaterials from renewable bioresources as well as CO_2_ and low-cost one carbon compounds through more sustainable bioprocesses using synthesized biosystems [8–10]. From an application point of view, this research belongs to industrial biotechnology, which is often referred to as the third wave of biotechnology after medical and agricultural biotechnology. As one of the most promising technologies, industrial biotechnology has the potential to address today’s great challenges such as climate change, environmental pollution, and food and resource shortage [11].

One of the key foundational technologies supporting synthetic biology studies is computing. The latest technological developments in computational hardware and software not only make it possible to store, manage, and share large amounts of biological data but also provide new and powerful algorithms for processing and analyzing the data for knowledge discovery, model creation, and computational design. In recent years, artificial intelligence (AI) reemerged as a hot topic not only in computer science but also in the general public domain, which was attributed to the development of AlphaGO by Google [12]. AI, or more generally including conventional machine learning (ML), is particularly suitable for detecting complex relationships within a large amount of data. AI algorithms, combined with the wealth of biological data, hold great potential for data-driven new biological discoveries and applications.

Recently, AI was adopted for many powerful applications in bioscience such as disease diagnosis, drug development, and protein structure prediction [13–16]. In this review, we will focus on the application of data-driven methods in industrial biotechnology for the design and construction of novel enzymes and artificial cell factories [17–21]. In this review, we will first give a brief introduction to the available data related to industrial biotechnology and ML and AI algorithms. The emphasis will be on how the data-driven approaches have been applied to address various biological problems such as prediction of function and physiochemical properties of proteins, design of nonnatural enzymes and biosynthesis pathways, or the optimization of metabolic engineering strategies. We will also discuss how the data-driven approaches should be integrated with approaches based on mechanistic insights to help us better understand and interpret the predictions.

## 2. Biological Data and Databases Related to Industrial Biotechnology

### 2.1. Data on Enzymes/Proteins

The key parameters affecting the industrial application of enzymes are the specific activity, substrate affinity, selectivity, and stability. All these functional parameters are ultimately determined by protein sequences that form complex 3D protein structures. Various genome sequencing projects have produced sequence data for a huge number of enzymes which can be easily searched and downloaded from various resources such as NCBI and EBI. In addition to sequences, protein structure data is extremely important for the design and engineering of proteins to achieve target properties. Although homology modeling algorithms are available to predict protein structure from sequences, the prediction accuracy is still far from perfect. Labor-intensive and costly experimental methods remain the standard way to determine protein structure. Currently, less than twenty thousand of the enzymes in UniProt [22] have structural data available in PDB [23], the protein structure database. For the quantitative functional parameters more relevant to industrial application, there are even fewer data available. Only about 7000 enzymes have kinetic information available in UniProt, and most of them are from a small number of well-studied model organisms such as humans and *E. coli*. For experimentally measured quantitative parameters, BRENDA (BRaunschweig ENzyme DAtabase) [24] is a very useful database with manually collected and curated data from published papers. For example, 562 enzymes from *E. coli* have reported specific activity values in BRENDA (578 have turnover numbers and 861 have Km values). In addition, the BRENDA database contains many other important data relevant for industrial applications such as enzyme stability, optimal pH, and temperature. A list of useful databases and the types of data available can be seen in Table 1.

**Table 1:** Databases with data relevant to industrial biotechnology.

Database	Web address	Data types
UniProt [22]	http://www.uniprot.org	A comprehensive, high-quality, and freely accessible resource of protein sequence and various functional and structural data
PDB [23]	http://www.rcsb.org	Biological macromolecular structures
KEGG [25]	https://www.genome.jp/kegg	A comprehensive database on genes, genomes, metabolic reactions, and pathways
BRENDA [24]	http://www.brenda-enzymes.info	A comprehensive enzyme database with quantitative information on enzyme kinetics, stability, optimal pH, temperature, etc.
BioCyc [26]	http://biocyc.org	Data on metabolic reactions and pathways in various organisms
BiGG [27]	http://Bigg.org	A collection of high-quality genome-scale metabolic models
GEO [28]	https://www.ncbi.nlm.nih.gov/geo	Database of publicly available gene expression datasets
ArrayExpress [29]	https://www.ebi.ac.uk/arrayexpress	Gene expression database
PAXdb [30]	http://pax-db.org	Protein abundance data for various organisms

### 2.2. Data on Metabolic Pathways and Networks

The conversion of a substrate into a valuable product often requires a series of reactions catalyzed by a number of enzymes. These successive reactions form a metabolic pathway. The metabolic network of an organism usually contains hundreds or thousands of enzyme-catalyzed reactions, which utilize different substrates to produce the cellular building blocks and many other metabolic products. The wide availability of genome sequences makes it possible to reconstruct genome-scale metabolic network models (GEMs) for a great number of organisms [31]. In the last two decades, hundreds of high-quality GEMs have been reconstructed and applied to simulate growth rates, determine minimal media and substrate usage profiles, predict essential genes, calculate optimal pathways, and design metabolic engineering strategies [32]. In addition to the data on genes and enzymes, data on metabolites and reactions are also required for metabolic network analysis. Table 1 lists a number of databases (KEGG, BioCyc, and BiGG) with information on metabolites/compounds and biochemical reactions, containing various types of data such as compound formulae, structures, and reaction thermodynamics.

### 2.3. Condition-Specific Data

The above-discussed data such as sequences, structures, and kinetic parameters are all static, i.e., they are fixed properties of a protein or reaction. As living systems, cells use complex regulatory networks to control the levels of proteins/metabolites at different growth stages and under different conditions. Dynamic omics data such as transcriptomics (mRNA levels), proteomics (protein levels), metabolomics (metabolite levels), and fluxomics (reaction rates) are also very important for the investigation of mechanisms behind cellular behaviors. In industrial biotechnology, it is also becoming very common to carry out dynamic omics analysis to study the time-course changes in a fermentation process or compare the expression patterns between a production strain and its wild-type parent. Unfortunately, these valuable data are often not deposited in databases. Moreover, a lack of standardized metadata describing the conditions relevant for the omics data makes it difficult to compare data produced by different research groups. Nevertheless, a few databases on transcriptomics (including those measured by microarray technology) and proteomics that mainly collect information from the literature are available [28–30] and listed in Table 1 (GEO, ArrayExpress, and PAXdb).

### 2.4. General Problems with Biological Data and Data Preprocessing

For quality data-driven studies, the data needs to be abundant, high quality, and well organized. Although a large amount of data available in databases makes it possible to carry out data-driven studies, great efforts still need to be made to further improve the quantity and quality of data to make them more accessible to analysis. Major problems and ways to address them are briefly discussed below.

(a) Data organization: By data organization, we mean if the data in one database are organized in a good way to capture all related information unambiguously. For example, BRENDA is the most important database of manually curated enzyme kinetic parameters from the literature [24]. However, the data is organized based on EC numbers, and in case that there are two enzymes with the same EC number (isoenzymes), it is difficult to know which specific enzyme was used to measure the kinetic parameters
(b) Data integration: Individual databases are often focused on certain specific topics and not comprehensive enough to answer a real biological question. It is quite common that integration of data from different databases is required, but the databases are not fully interlinked. Even though major databases provide cross-links to other databases, these cross-links are often not complete or are broken due to a lack of updates. A proper entity ID is the key for cross-linking. Unfortunately, different databases (and publications) tend to use different IDs for the same entity. For example, the same *argA* gene of *E. coli* has several IDs (e.g., b2818, EG10063, ECK2814) used in different databases as well as various synonyms (used more often in published papers). The situation is even worse for mapping compounds and reactions due to their hierarchical relationships. For example, glucose, D-glucose, alpha-D-glucose, and beta-D-glucose may be mixed up in different databases. ID mapping tools such as that provided by UniProt (https://www.uniprot.org/uploadlists/) for gene mapping and MetanetX [33] for compound mapping partially address this problem, but data integration remains a headache for data-driven studies
(c) Data quality/inconsistency: The data in databases may be generated/collected in different ways or through different curation and quality control processes. For example, in the UniProt database, the manually annotated and reviewed data (less than 1%) are saved in SwissProt, while the computationally annotated data are in TrEMBL [22]. For enzyme kinetics, different values might be reported by different researchers due to the variety of measurement protocols for the same enzyme, leading to data inconsistencies. Certain inconsistencies are caused by low biological repeatability due to the complexity of biosystems, especially high-throughput measurement data for living cells. Noise reduction to improve data quality is an important step in data-driven studies. Typically, the denoising approaches consist of two major steps: noise identification and noise handling. There exist three main categories of techniques for noise identification: ensemble techniques, distance-based algorithms, and learning-based techniques [34]. For noise handling, the simplest way is ignoring the noisy data, but it comes at the cost of loss of data precision. To enclose more information, the commonly used ways are data cleaning techniques, and lots of methods are developed for data cleanings, such as data filtering and multiscale denoising [35]
(d) Data standardization: Automatic data preprocessing and analysis by programming are essential for data-intensive studies. To this end, all data should be represented and stored in standard formats so that they can be easily shared and read by various software tools. Scientists have developed certain standard formats to represent biological data, such as SBML for kinetic and metabolic models [36], or SBGN for the graphical representation of biological networks. Another aspect of data standardization is the use of metadata to attach information related to the data, such as the strain information, sampling time, and method for a set of transcriptomic data. This is particularly important for condition-specific data, as these can only be correctly interpreted together with the metadata. To this end, the scientific community proposed standard guidelines such as MIAME (Minimum Information About a Microarray Experiment) [37] to ensure that necessary metadata is submitted together with the main datasets
(e) Uneven distribution of data: As discussed in previous sections, there are different types of biological data, and certain technologies such as sequencing-based genomics, metagenomics, and transcriptomics generate big data. However, most small-scale experiments, such as those related to cellular phenotypes and macromolecular structures, generate comparably small datasets. The data availability for various organisms is also very different. Most of our biological knowledge and data are actually obtained by studying a small number of model organisms such as humans, yeast, and *E. coli*. In addition, our expertise on different subsystems is also unevenly distributed. For example, kinetic and structural information is often available for enzymes in the central pathways, while it is patchy for other pathways. Using existing data to computationally predict the missing data is another important application of data-driven studies. We will show a few such examples in Section 4

## 3. Data-Driven Algorithms

**Figure 1:**
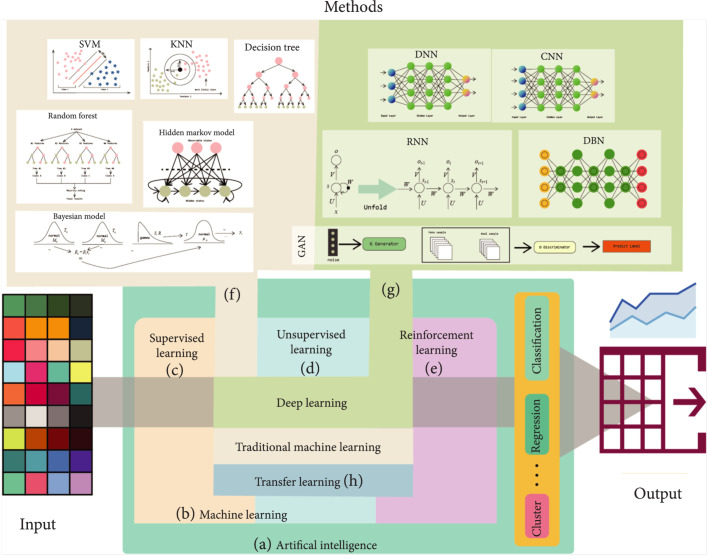
Representation of the AI, ML, and DL frameworks for application in data-driven studies. AI (a) is the broad science of mimicking human abilities, and ML (b) is a specific subset of AI. ML can generally be grouped into supervised (c), unsupervised (d), and reinforcement learning (e) according to learning tasks. Based on learning techniques, ML can be categorized into traditional ML (representative methods as shown in (f)), deep learning (representative methods as shown in (g)), and transfer learning (h).

The main objective of data-driven studies is to use available data as inputs to predict functions/properties of new systems or under new conditions. Various computational algorithms have been developed to learn the input-output relationships from available data. ML usually refers to the traditional machine learning algorithms, while the recently developed deep learning (DL) methods are often classified as AI algorithms. However, these terms are often used in similar contexts, and ML is also regarded as a form of AI. A general representation of the principle and the algorithms can be seen in Figure 1. In the following section, we will briefly introduce the concepts and algorithms.

### 3.1. Brief Introduction of ML Algorithms

ML is often used to recognize patterns in data and make predictions once new data arrives. ML algorithms are organized into a taxonomy based on the desired inputs and outputs of the algorithms [38]. There are many ways to categorize these algorithms, but conventionally, the ML approaches can be broadly categorized into supervised learning, unsupervised learning, and reinforcement learning [39].

In supervised learning, an algorithm is presented with labeled data, which means that each item of the input data is associated with a correct response, such as numeric values or string labels. The goal is to approximate the mapping function to predict the output from new input data. Supervised learning problems include classification (determining what group a given input belongs to) and regression (predicting a quantitative production). For example, the prediction of the metabolic pathway associated with a metabolite is a classification problem. At the same time, the prediction of the specific activity of an enzyme from its sequence and structure features is a regression problem. Routinely used supervised methods include Artificial Neural Networks (ANNs) and their variants, Multi-Layer Perceptron (MLP), Linear Regression (LR), Support Vector Machine (SVM), K-Nearest Neighbor (KNN), Decision Tree (DT), Random Forest (RF), Hidden Markov Model (HMM), and Bayesian model-based methods [40].

Unsupervised learning resembles the methods humans use to deduce that certain objects or events are from the same class, such as observing the degree of similarity between objects (no data label is required). Unsupervised learning algorithms include clustering and dimensionality reduction. Clustering is similar to classification but groups the objects based on some similarity in input features rather than learning from prior knowledge on classification. There are different clustering methods, such as partitional, hierarchical, grid, and density-based. Dimensionality reduction transforms data from a high-dimensional space into a low-dimensional space so that the low dimension variables retain some meaningful properties of the original data, ideally close to its intrinsic size. Dimensionality reduction is prevalent in fields that deal with large numbers of observations (variables) and a small number of instances (samples) [41]. Omics data analysis is a typical case where we have data for thousands of genes but only a few data points. General unsupervised algorithms include Principal Component Analysis (PCA), Singular Value Decomposition (SVD), Markov Chain Monte Carlo (MCMC), Linear Discriminant Analysis (LDA), and Non-negative Matrix Factorization (NMF) [40].

Reinforcement learning is relatively distinct from supervised and unsupervised learning. Reinforcement learning differs from supervised learning in not requiring labeled data. Instead, the focus is on balancing exploration (of uncharted territory) and exploitation (of current knowledge) [42]. In reinforcement learning, the algorithm learns a policy of how to act based on observation of the world. Every action impacts the environment, and the environment provides feedback that guides the learning algorithm.

### 3.2. Recent Development of DL Approaches

DL is a specific type of ML that has been particularly successful in the past decade. The most important difference between DL and traditional ML is its performance as the scale of data increases. With small datasets, DL algorithms do not perform that well because they need a large amount of data to understand them. The core concept of DL is to learn data representations through increasing abstraction levels, and its success lies in the careful design of the neural network architecture. It enables a neuronal system to learn complex representations directly from the raw data, making it a powerful method for dealing with problems in many disciplines [43]. A large number of explicitly designed new network structures are proposed in a high-frequency manner. These networks are mainly based on Artificial Neural Network (ANN), Deep Neural Network (DNN), Convolutional Neural Network (CNN), Recurrent Neural Network (RNN), or Generative Adversarial Network (GAN) architectures, and their variants [14, 44].

ANNs are computational processing systems that mimic the way biological nervous systems operate. ANNs usually load a multidimensional vector into the input layer and map it to the hidden layers. The hidden layers will then make decisions based on the previous layer and weigh how a stochastic change within itself degrades or improves the final output, and this change is referred to as the process of learning. DNN is an ANN with multiple layers between the input and output layers. DNN is typically a Feed-Forward Network where data flows from the input layer toward the output layer without flowing backward. The network weights are obtained by supervised learning through backpropagation with hidden information datasets.

CNN is a type of DL model for processing data with grid patterns, such as images, which is inspired by the organization of the visual cortex of animals and designed to automatically and adaptively learn spatial hierarchies of features, from low- to high-level patterns. CNN is a mathematical construct typically composed of convolution, pooling, and fully connected layers. The convolution and pooling layers perform feature extraction, whereas the fully connected layer maps the extracted features into the final output [45]. RNN considers that what has happened in the past is likely to impact what happens in the future, which is typically suitable for modeling sequential data. GAN provides a way to learn deep representations without extensively annotated training data. They achieve this by deriving backpropagation signals through a competitive process involving a pair of networks. This technique learns to generate new data with the same statistics as a given training set. For example, a GAN trained on photographs can generate new pictures that look superficially authentic to human observers. The core idea is based on “indirect” training through the discriminator, which itself is also being updated dynamically. This basically means that the generator is not trained to minimize the distance to a specific target but rather to fool the discriminator. This enables the model to learn in an unsupervised manner [46].

## 4. Application of Data-Driven Approaches in Biomanufacturing

### 4.1. Data-Driven Approaches in Enzyme/Protein Design

Natural enzymes have long been used to catalyze a wide variety of chemical reactions, but their properties and catalytic capacity are often inadequate to meet the needs of industry. Therefore, scientists have been developing new techniques to engineer and build enzymes with properties that meet industrial production requirements [47]. On the other hand, they are also building enzymes that do not exist in nature by further enhancing the development of protein folding control techniques [48, 49]. In addition to classical rational design and directed evolution approaches, ML and DL methods have been increasingly applied to help scientists predict protein structures and improve protein functions [20, 50–52].

Traditionally, quantum mechanics, molecular mechanics, and QSAR have been the main calculation tools used to study proteins’ structural and functional properties. However, the huge potential conformational space makes it almost impossible to find the overall lowest energy conformation of a protein by space traversing using quantum mechanics or molecular mechanics. Rational enzyme design efforts have been mainly limited to the localization of the reaction center of the enzyme. In contrast, structural modification of the broader regions of the enzyme could only be achieved by random mutation or directed evolution techniques using extensive experimental protocols that exploit the powerful natural ability of the organism to evolve. However, with the accelerated accumulation of protein sequence and structural information in recent years, data-driven enzyme design based on ML and DL techniques is showing strong potential, and four main directions are discussed below.

The first research direction is to analyze the relationship between the sequence and properties of an enzyme. ML techniques are applied to guide the selection of initial evolutionary routes for directed evolution [53, 54]. DL techniques are widely used to analyze and annotate the function and properties of enzymes, such as the prediction of enzyme EC number [55], enzyme activity [56], substrate selectivity [57], thermal stability [58], and solubility [59, 60]. Moreover, ML has been gradually applied to guide enzyme modification [61–63]. For example, Ryu et al. developed DeepEC, a deep learning tool that can predict enzyme EC numbers from protein sequences [55]. They compiled a dataset containing 1,388,606 expert-curated reference protein sequences and 4669 EC numbers from the UniProt database to train the deep neural networks. The prediction accuracy and speed were improved compared with other computational prediction tools. DeepEC was also shown to be more sensitive in predicting the effects of binding site mutations and could improve the accuracy of homology-based annotation [55]. The improved EC number prediction can also help refine the reconstructed metabolic network by gap filling with new enzyme functions. Thermal stability is an important protein property that greatly impacts the cost of biomanufacturing. Chen et al. developed the ML method iStable 2.0 for stability prediction by integrating 11 sequence- and structure-based prediction tools and adding protein sequence information as features. The integrated ML model had a higher Matthews correlation coefficient than individual prediction tools [64].

The second area of research, protein structure prediction, is a key basic science problem that researchers have been working on for a long time [65]. Recently, the development of AlphaFold and AlphaFold2 by Google scientists has led to a new era of accurate protein structure prediction, allowing scientists to use protein sequences to predict enzyme structures for accurate rational design [66, 67]. This has ameliorated the problem that structure analysis lags behind the application needs.

The third research direction is to enhance the computational accuracy and extend the sampling space of traditional algorithms to compensate for the deficiencies of physical rule-based evaluation systems. Several studies have used ML and DL in conjunction with traditional rational design methods to improve the accuracy of traditional algorithms in protein structure optimization [67]and protein-small molecule and protein-protein binding energy calculations, as well as the sampling range of binding conformations [68, 69].

The fourth research direction is to design enzymes with novel functions [70]. The ab initio design of new enzymes can be seen as a reverse operation of protein structure prediction [71]. The design process can be divided into two parts: finding or constructing a protein backbone that meets the reaction requirements and selecting the proper sequence to achieve the protein function based on the backbone. The Rosetta software developed by David Baker’s research team has been widely used for the construction of protein backbones [72]. A series of sequence design algorithms recently developed based on DL have also demonstrated their application potential [52, 73]. An effective combination of the two approaches will greatly promote new enzyme design technologies.

### 4.2. Application of ML in Cell Engineering

Data related to living cells are more complex than those related to isolated proteins. From the aspect of input data, the abundant sequence data is far from enough to predict cellular behaviors. Many other factors are important, but data quality is also poor with fewer data available. From the aspect of output, an engineered cell factory needs to possess multiple desirable properties to make the bioconversion process economically viable, such as high product titer, rate, and yield (TRY). Therefore, unlike data-driven protein design, which often has one clearly defined output as its goal, data-driven cell studies are multiple-input multiple-output (MIMO) problems by nature. Due to the complex nonlinear relationships between the inputs and outputs, it is not straightforward to train multiple individual models for different outputs and then combine them together for reliable collective prediction. Despite these challenges, scientists have made great efforts in recent years to use data-driven approaches in cell engineering by focusing on particular problems where clearly defined inputs and objectives are available.

#### 4.2.1. ML for Metabolic Network Model Reconstruction

Genome-based metabolic network models (GEMs) are important tools in the study of cellular phenomena and the design of metabolic engineering targets for creating artificial cell factories. Many efforts have been made to reconstruct high-quality GEMs to improve model prediction accuracy. Recently, ML methods were also applied to address various issues in GEM reconstruction, such as determining the metabolic function of new genes, filling gaps in pathways, or adding new constraints [21, 74, 75]. A draft GEM computational reconstructed from genome often contains many gaps due to incomplete and inaccurate enzyme gene annotation mainly based on sequence similarity. Gap filling to add the missing reactions and their corresponding genes is a necessary step to obtain a reliable GEM. Though several gap-filling algorithms have been developed, most of them only find the candidate reactions from reaction databases but fail to identify the possible enzyme genes for the gap-filling reaction. The AI methods used for the prediction of the EC number of a protein discussed in Section 4.1 can be used to predict more enzyme genes and thereby possibly link new enzyme genes with the gap-filling reactions. Recently, Luo et al. adopted DeepEC to obtain more EC numbers in reconstructing a GEM of *Shewanella oneidensis* MR-1 and the resulted model has much more reactions and genes than previously published models [76]. It should be noted that the relationships between EC numbers and reactions are multiple to multiple, namely, multiple reactions can be catalyzed by enzymes with the same EC number, and the same reaction can be catalyzed by enzymes with different EC numbers. Particularly for those EC numbers with broad substrate specificity, such as 1.1.1.1 (alcohol: NAD+oxidoreductase), the exact alcohol substrate for a specific enzyme protein cannot be determined explicitly by the EC number. In one organism, a protein annotated with this EC number may catalyze the oxidation of 1-propanol, while in another organism, a protein with the same EC number may catalyze a reaction using 2,3-butanediol. Therefore, it is desirable to directly predict the responses rather than via the EC numbers to reconstruct a more reliable metabolic network. However, this requires a comprehensive ontological classification of reactions based on mechanisms. Although databases like Rhea [77] tried to include information on reaction relationships, a more helpful gene ontology-like classification of responses is still missing. Researchers therefore still must rely on EC number predictions first and use an extra step to add reactions into the network based on reaction-EC relationships. An alternative approach is to focus on specific enzymes rather than all EC numbers. For example, Cai et al. used reaction fingerprints and ML methods to predict enzymatic reactions of oxidoreductases and hydrolases [78]. Such approaches have the potential to predict the exact substrate of an enzyme, but a prescreening step (that can often be solved as a 0-1 binary classification problem) is required to ensure that the protein belongs to a specific class of enzymes. There are also several ML applications in predicting the pathways of a protein instead of the exact reaction [79, 80]. This pathway prediction can help find candidate enzymes for gap filling in a pathway, but it is still necessary to determine the exact reaction if multiple gaps are present.

#### 4.2.2. Design of Non-Natural Metabolic Pathways

Although the major metabolic reaction databases contain tens of thousands of known reactions, there are still many compounds (in particular, many valuable natural products) whose synthetic pathway is unknown or even nonexist. Even for the compounds with known biosynthetic pathways, it is desirable to design novel non-natural pathways which may have a higher yield or fewer steps than the natural pathways. Retrosynthetic methods that start with the desired chemical and suggest a set of chemical reactions that could produce it from specific precursors have been developed to design novel pathways [81, 82]. One main problem in retrosynthetic pathway design is the huge number of possible reaction combinations generated by high-level reaction rules. One critical task is to use the optimization or heuristic methods to choose the right combination of reactions, which are more likely to be successfully constructed. Recently, Koch et al. [81] proposed a new method called RetroPath RL, which explores the retro-biosynthetic space using an artificial intelligence-based approach relying on the Monte Carlo Tree Search reinforcement learning method. They validated the method using a dataset of 20 manually curated experimental pathways as well as a larger dataset of 152 successful metabolic engineering projects. This proves the usefulness of data-driven methods in the design of non-natural pathways. Instead of predicting the exact reactions for the synthesis of a compound, Baranwal et al. [83] aimed to predict the metabolic pathway with which a given compound is likely to be associated since the metabolites in that pathway could be suitable substrates for the synthesis of the new compound. They used a hybrid ML approach consisting of graph convolutional networks to solve the task as a classification problem. They extracted relevant shape features directly from SMILES representations of molecular structures. The trained model correctly predicted the respective metabolic pathway class for 95.16% of tested compounds.

Prediction of possible biosynthesis pathways is just the first step in designing a non-natural pathway, and it is also necessary to identify promiscuous enzymes that can catalyze the novel biochemical reactions in the pathway. Protein engineering to improve the selectivity/activity of the new reactions is also necessary to optimize the novel pathway beyond the capabilities of natural pathways. In addition to the bioinformatic methods used for enzyme screening and protein structure analysis, ML methods can also be used to design proteins with desired substrate activities as discussed in 4.1.

#### 4.2.3. Prediction of Kinetic Parameters

As discussed in Section 2, the coverage of data related to enzyme kinetics is very low and often focused on a small set of pathways or a few important enzymes for a limited number of well-studied model organisms. This is ok for developing a kinetic model of a metabolic pathway but far from enough for whole cell model. In recent years, enzyme-constrained models (ECMs), which integrate enzymatic constraints into GEMs, have been shown to be more powerful and reliable in simulating/predicting cellular phenotype [84–86]. ECMs require a whole network level coverage of enzyme kinetic parameter values for accurate prediction. Unfortunately, even in model organisms like *E. coli*, there is no measured kinetic data for more than half of the enzymes. As there is no high-throughput method for enzyme kinetics and the traditional methods are costly and time-consuming, it is desirable to use data-driven strategies to computationally predict the kinetic data. Mellor et al. used a semi-supervised Gaussian process regression model to predict the substrate affinity of proteins (Km values) [87]. Input signatures for the model were defined based on chemical transformation rules using extended connectivity fingerprint descriptors. Their model prediction was validated experimentally by correctly finding the enzymes catalyzing the reactions associated with a newly identified metabolite in *E. coli*. In a recent study, Heckmann et al. applied ML methods to successfully predict catalytic turnover numbers (kcat) for *E. coli* enzymes based on integrated data on enzyme biochemistry, protein structure, and network context [88]. Using this approach, they obtained kcat values for all enzymes that catalyze reactions in the *E. coli* GEM and developed an enzyme-constrained model showing significantly higher accuracy in predicting quantitative proteome data than previous approaches.

Scientists have also developed data-driven approaches to directly simulate metabolic behaviors without using a kinetic model. Costello et al. showed that supervised ML can directly deduce the relationship between metabolites and enzymes from time series of protein and metabolite concentration data [19]. The inputs were the exogenous pathway proteins and metabolite concentrations, and the response was the rate of change of the metabolite. This approach is beneficial when only limited time-course data are available due to the high cost of performing multi-omics experiments. They used data augmentation to increase the amount of available data for model training, and the final model outperformed the kinetic model.

#### 4.2.4. Pathway/Strain Optimization

It is difficult to predict the optimal engineering strategies for constructing an industrial strain using data-driven approaches due to a large number of possible targets and genetic operations. However, these approaches can still be helpful if we focus the engineering targets on particular pathways/modules. For example, Karim et al. used deep neural networks to optimize a six-step pathway for cell-free butanol production [89]. Six enzyme homologs at three different concentrations for each step resulted in over 34 million pathway combinations, and data-driven approaches can be applied to predict the optimal combination from a small experimental dataset. They used the enzyme homologs and their corresponding concentrations as the input for the neural network, with a defined TREE score combining the titer, rate, and enzyme expression as the output. The predicted combination from the neural network model improved TREE scores over fourfold compared to the initial pathway.

In cellular systems, it is not possible to directly change the enzyme concentrations as is possible in cell-free systems. The enzyme (gene) expression level is often regulated using different promoters or RBSs. Choosing the optimal promoter/RBS combinations in a pathway by ML has also been used in strain optimization. Zhou et al. used neural networks to improve a 5-step pathway for violacein production by selecting promoter combinations using an initial training set of only 24 strains [90]. The predicted strain improved the violacein titer 2.4-fold after only 1 DBTL iteration. Similarly, Opgenorth et al. used an ensemble of four different models (Random Forest, Polynomial, Multi-Layer Perceptron, TPOT Meta-Learner) to optimize a 3-step pathway for dodecanol production by predicting the optimal RBS combinations [91]. By combining RBS and a promoter library, Hamedi et al. were able to regulate the gene expression at 24 distinct levels with a ~1000-fold dynamic range. Using initial data generated by a fully automated robotic platform, they quickly improved lycopene synthesis in *E. coli* using ML models [92]. Various ML models were also used to guide the improvement of limonene production in *E. coli* [93] and tryptophan production in *S. cerevisiae* [94]. These studies highlight the potential of ML in pathway optimization. To enable broader use of data-driven pathway optimization, Radivojevic et al. developed the Automated Recommendation Tool (ART) [95], which is specifically tailored to the needs of metabolic engineering.

## 5. Discussion

Data-driven approaches have promising application prospects in various research fields related to biomanufacturing, ranging from enzymes to whole cells. With the availability of more standardized biological data and the development of new specifically tailored algorithms, these data-driven approaches will play important roles in future studies. However, to realize the full potential of these approaches in addressing real-world problems, several obstacles still need to be overcome. One fundamental problem of data-driven methods is the lack of interpretability. By nature, ML and particularly DL algorithms are based on black-box models. The algorithm generates a predicted output but it remains unknown why the model produces this output from a given set of input values. This is in great contrast with mechanistic models, yet understanding the mechanisms underlying complex and fascinating behaviors of biosystems is a major motivation for most scientists. Therefore, even though data-driven methods may have strong predictive power to solve real-world problems, they cannot satisfy the desire to pursue new knowledge. Consequently, people are also unlikely to be very confident of the predictions of a black-box model. Moreover, it is very difficult to troubleshoot the data-driven model if the model prediction is inconsistent with the experimental test. The only options are to retrain the model with added new data or adjust the parameters of the models (e.g., add more layers or nodes in a DL model). However, like other data-fitting methods, there is a risk of overfitting. Especially DL algorithms are so powerful in capturing the nonlinear input-output relationships that they can practically always deliver good fitting for existing data [96, 97], but do not represent the true cause-effect relationships in the biosystem. For the optimization of biomanufacturing processes, the expected output of an enzyme property or cell property is highly likely to be beyond the boundary of the existing data. The power of the model is therefore more likely to lie in choosing a small set of inputs (rather than a huge number of combinations) for experimental verification instead of making precise quantitative predictions. Researchers must therefore be very careful in interpreting the results of data-driven models to avoid overfitting or misinterpretation akin to the misuse of *p*-values in statistical analysis of biological data by many researchers [98].

One possible way to address the noninterpretability problem is to combine data-driven approaches with mechanistic models. For example, scientists may focus on a small set of engineering targets for strain optimization in data-driven studies to avoid combinatorial explosion, and a mechanistic model can help in choosing a proper set of targets that are more likely to be effective. This can be achieved using methods such as metabolic control analysis [99] and various target identification methods based on genome-scale metabolic network analysis [100, 101]. In a recent study, Zhang et al. used a genome-scale model to pinpoint five genes as engineering targets for improving tryptophan production in *S. cerevisiae* [94]. Each gene was expressed at six different levels using separate promoters, after which they trained ML models using data points derived from >500 different strain designs and used model predictions to guide the strain design process. The tryptophan titer and productivity of the best strain were improved by up to 74 and 43%, respectively. Notably, all five gene targets were in central metabolic pathways rather than the tryptophan synthesis pathway. Without prior target selection based on a mechanistic model and biochemical knowledge, there would be too many possible modification targets requiring large amounts of experimental data to train the model, significantly increasing the time and cost for strain optimization. In a recent study, Czajka et al. proposed a new platform to integrate knowledge mining, feature extraction, genome-scale modeling, and ML for more realistic engineering target prediction and applied it in the design of an engineered *Yarrowia lipolytica* strain with improved product titers [102].

Another major problem of data-driven methods is the so-called “curse” of dimensionality, caused by the exponential increase in the amount of data needed to support results with the dimensionality of the input. Unfortunately, most biological data, especially omics data, suffers from this problem. There are many measured variables (genes, metabolites, etc.) but few instances (sometimes only at two different conditions or for two genotypes). Even after integrating data from databases, the number of instances can reach only about a thousand for model organisms, and much less for other organisms, while reliable ML models normally require around 100 instances per 5–10 variables [19]. Therefore, there is a severe lack of data for system-level analysis with thousands of genes. Due to the still high cost of omics data generation, it is essential to reduce the dimensionality of the problem for data-driven studies. Most studies described above focused on particular problems considering a small set of input variables. Choosing a small number of important inputs from thousands of available ones is key to the success of ML models using limited data. This requires experience and rich knowledge of the biological system, which is where mechanistic models can help. Data-driven approaches should be combined with prior knowledge of the studied biosystem to realize its full potential in developing novel enzymes and strains for biomanufacturing.

## 6. Conclusion

Data-driven methods offer an opportunity to make reliable predictions without the need of building mechanistic models. This is particularly useful for complex biosystems of which our knowledge is very patchy. This review summarized the application of data-driven methods for the development of synthetic cell factories. We show examples on protein function prediction, metabolic model reconstruction, kinetic data estimation, nonnatural pathway design, and strain optimization. We propose the integration of data-driven approaches with mechanistic model approaches to speed up the development of synthetic cell factories while at the same time improve our knowledge on the cells.
